# Ventral Attention Network Correlates With High Traits of Emotion Dysregulation in Community Women — A Resting-State EEG Study

**DOI:** 10.3389/fnhum.2022.895034

**Published:** 2022-05-26

**Authors:** Francesca Fusina, Marco Marino, Chiara Spironelli, Alessandro Angrilli

**Affiliations:** ^1^Padova Neuroscience Center, University of Padua, Padua, Italy; ^2^Department of General Psychology, University of Padua, Padua, Italy; ^3^Department of Movement Sciences, Research Center for Motor Control and Neuroplasticity, KU Leuven, Leuven, Belgium; ^4^IRCCS San Camillo Hospital, Venice, Italy

**Keywords:** functional connectivity, EEG, emotion dysregulation, resting state, default mode network (DMN), ventral attention network (VAN)

## Abstract

In recent years, many studies have focused on resting-state brain activity, and especially on functional connectivity (FC), an approach that typically describes the statistical interdependence of activity in distant brain regions through specific networks. Our aim was to study the neurophysiological correlates of emotion dysregulation. Therefore, we expected that both the Default Mode Network (DMN), and the Ventral Attention Network (VAN) would have been involved. Indeed, the latter plays a role in the automatic orienting of attention towards biologically salient stimuli and includes key regions for emotion control and modulation. Starting from a community sample of 422 female students, we selected 25 women with high traits of emotion dysregulation (HD group) and 25 with low traits (LD group). They underwent a 64-channel EEG recording during a five-minute resting state with eyes open. Seed-based FC was computed on the EEG Alpha band (8–13 Hz) as a control band, and on EEG Gamma power (30–50 Hz) as the relevant measure. The power within each network and inter-network connectivity (Inter-NC) was also calculated. Analysis of the EEG Gamma band revealed, in the HD group, higher levels of Inter-NC between the VAN and all other resting-state networks as compared with the LD group, while no differences emerged in the Alpha band. Concerning correlations, Alpha power in the VAN was negatively correlated in the HD group with affective lability (ALS-18 questionnaire), both for total score (*ρ* = –0.52, *p*_FDR_ < 0.01) and the Depression/Elation subscale) *ρ* = −0.45, *p*_FDR_ < 0.05). Consistent with this, in the Gamma band, a positive correlation was found between VAN spectral power and the Depression/Elation subscale of ALS-18, again in the HD group only (*ρ* = 0.47, *p*_FDR_ < 0.05). In conclusion, both resting state FC and network power in the VAN were found to be related to high emotion dysregulation, even in our non-clinical sample with high traits. Emotion dysregulation was characterized, in the EEG gamma band, by a VAN strongly connected to all other networks, a result that points, in women prone to emotion dysregulation, to a strong automatic orienting of attention towards their internal state, bodily sensations, and emotionally intense related thoughts.

## Introduction

The activity of the brain at rest, also called *intrinsic activity*, is thought to be responsible for the largest fraction of neural energy consumption and maybe even more significant than the activity elicited by a specific stimulus as an index of global brain processing capacities (Raichle and Mintun, 2006). For this reason, in recent years, a growing body of research has been dedicated to resting state brain activity, with the majority of studies relying on neuroimaging techniques, including blood oxygenation level-dependent (BOLD) functional Magnetic Resonance Imaging (fMRI; Van Den Heuvel and Pol, 2010; Lee et al., 2013). fMRI provides an excellent spatial resolution but has a limited temporal resolution (Ahmad et al., 2015), which might be associated with participant discomfort due to the claustrophobic recording space and the scanning noise (Gaab et al., 2008; Glover, 2011). Lastly, with fMRI participants lie down in a supine/horizontal position, thus assuming that cognitive processes are not affected by the participant’s posture, an assumption that has been recently confuted (Spironelli et al., 2016; Spironelli and Angrilli, 2017). Of particular interest is magnetoencephalography (MEG), a technique even more precise than EEG, but more expensive and limited to very few equipment in the world. MEG has been successfully used to assess Resting State Functional Connectivity (rsFC) alteration in psychiatric disorders (Alamian et al., 2017). This review of five investigations carried out on Major Depression and Bipolar patients revealed a good convergence of MEG with EEG and fMRI methods in the assessment of the altered connectivity in psychiatric disorders. Both MEG and EEG, compared with fMRI provide a direct measure of neural activity at high temporal resolution (i.e., in the order of milliseconds) by detecting magnetic fields/scalp potentials (Ahmad et al., 2015). A further advantage of EEG is that it is silent and portable, allowing for greater flexibility than fMRI and MEG in a variety of experimental designs and conditions (Glover, 2011). EEG ensures ecological validity, meaning a condition which is more similar to a real-world setting, as participants usually sit upright in front of a computer screen rather than lying down in an unusual posture (e.g., Spironelli and Angrilli, 2017).

In addition to conventional EEG spectral analysis (Newson and Thiagarajan, 2019), which investigates changes in brain activity in specific areas, the past two decades have seen the rise of research focusing on changes in *functional connectivity* (FC), which is defined as the temporal correlation between the activity from different brain regions (Greicius et al., 2009). FC paradigms are often applied at rest (resting state functional connectivity; rs-FC), which is an especially advantageous approach given its task-independent nature (Gillebert and Mantini, 2013) and have led to the identification of several networks functionally organized in the resting brain (Damoiseaux et al., 2006; Fox and Raichle, 2007; Smitha et al., 2017) among which are the Default Mode Network (DMN), the Dorsal Attention Network (DAN), the Somatomotor Network (SMN), and the Visual Network (VN), all bilaterally distributed. Instead, the Ventral Attention Network (VAN) and the Language Network (LN) are lateralized, on the right and on the left hemispheres, respectively (Samogin et al., 2020).

Most of these networks are altered in several severe psychiatric illnesses, such as depression, and schizophrenia (Chen et al., 2017) and bipolar disorder (Damborská et al., 2019). From a clinical perspective, these functional alterations might lead to deficits in behavior, including the capacity of regulating emotions, which represents an important risk factor for the future development of clinical disorders. Indeed, emotion dysregulation is often defined as an excessive emotional reactivity coupled with a basic inability to appropriately modulate emotional responses coherently with environmental demands and impairment in returning to a baseline state (Gratz and Roemer, 2004; Ebner-Priemer et al., 2015).

To the best of our knowledge, no studies have yet specifically targeted emotion dysregulation and rs-FC, either using fMRI or EEG-derived measures of FC. Most studies have investigated emotion regulation either during tasks (Allard and and Kensinger, 2014; Morawetz et al., 2016) or in populations with long-standing diagnoses of psychiatric conditions, such as anxiety and depression (Lui et al., 2011; Klumpp et al., 2018; for a comprehensive meta-analysis on the topic, see Kaiser et al., 2015), bipolar disorder (Marino et al., 2021), addiction (Sutherland et al., 2012), and Borderline Personality Disorder (BPD; e.g., Xu et al., 2016; *see below*).

Concerning studies on emotion regulation, while the insights they provide are crucial in pinpointing the regions of interest that may also be involved in emotion dysregulation, it must be kept in mind that “*emotion dysregulation is not simply inadequate emotion regulation*” (Thompson, 2019). Indeed, while it is true that emotion dysregulation may arise when the individual adopts maladaptive emotion regulation strategies (for example, rumination or avoidance), as we earlier mentioned it also encompasses instances in which emotions are expressed or experienced as inappropriate for the context, or when they change too abruptly or too slowly, and when they return to a baseline state is impaired (Gratz and Roemer, 2004; Ebner-Priemer et al., 2015; Cole et al., 2017; Thompson, 2019). Moreover, some authors argue that the concept of emotion regulation is problematic from a scientific point of view, due to the lack of a direct, explicit index. It instead relies on the quantification of the absence of problematic behavior when the individual is presented with environmental challenges (Cole et al., 2004; Beauchaine, 2015) and involves, by definition, a specific action undertaken by the subject, for example, reappraisal, willful up-regulation, or down-regulation (Frank et al., 2014). For this reason, the vast majority of studies regarding FC and emotion regulation are not conducted during resting state, but are focused on specific tasks, which can heavily interfere with basic neurophysiological responses not related to the task. As was previously mentioned, resting state studies have the important advantage of being independent of specific paradigms (Banks et al., 2007; Frank et al., 2014; Uchida et al., 2015; Sarkheil et al., 2020). Another limiting factor of these studies concerns the fact that the main interest of the few that do examine rs-FC and emotion regulation is often not emotion regulation/dysregulation *per se*, but actually a specific overarching psychiatric condition. An example lies in studies concerning emotion regulation in Major Depressive Disorder, in which rs-FC is examined as a potential mediator between symptoms of maladaptive emotion regulation and depression (Lopez et al., 2018).

When analyzing the literature regarding extreme and clinical manifestations of emotion dysregulation, such as BPD, studies rely on quite heterogeneous methods, thus yielding inconsistent results. Considering fMRI studies only, a large variety of FC approaches have been employed, including Amplitude of Low-Frequency Fluctuation (ALFF), regional homogeneity (ReHo), and seed-based (Lei et al., 2017), independent component analysis (ICA; Wolf, 2011), and graph theory (Xu et al., 2016). Additionally, the great heterogeneity of the samples considered, in terms of age, gender, comorbidities, and pharmacological treatment has added further levels of potential confounds in this type of research (Visintin et al., 2016).

Taken together, these findings point towards disruptions in a fronto-limbic network which includes regions that are implicated in both the processing of emotions, such as the amygdala and the insula, and their regulation, from the frontal cortical regions (Ruocco et al., 2013; Krause-Utz et al., 2014); however, these results still have not identified a clear mechanism underlying emotional dysregulation and negative affectivity in BPD population.

While many studies have focused on DMN alterations in BPD patients (for a meta-analysis on the subject, see Yang et al., 2016), we suggest that examining connectivity patterns in other RSNs could unveil interesting insights into the mechanisms underlying emotion dysregulation. For example, the VAN, which comprises the right temporo-parietal junction (rTPJ) and the right inferior frontal gyrus (rIFG; Samogin et al., 2020), is important for the involuntary orienting of attention towards salient stimuli (Corbetta et al., 2008), and increased EEG Gamma (>30 Hz) activity in VAN regions was found when bottom-up attentional control arises after a distracting stimulus is presented (ElShafei et al., 2020). Attentional biases towards emotional stimuli, especially when negative, were found, for example, in individuals suffering from anxiety (Bar-Haim et al., 2007) or having experienced childhood abuse (Gibb et al., 2009), as well as in BPD patients. In this latter population, a key role was attributed to emotional stimuli that are relevant to the patients’ personal experience (Kaiser et al., 2016). A study by Mao et al. (2020) analyzed the relationship between attention, early life stress, and depression, and found positive correlations between early life stress and connectivity in the VAN with the DAN, the SMN, and the VN. Within-network connectivity of the VAN was also found to mediate the relationship between early life stress and depression. Considering these results and the VAN’s role in the involuntary orienting of attention towards salient stimuli (Corbetta et al., 2008), the authors infer that attention biases might depend on VAN function (Mao et al., 2020).

Moreover, the VAN is especially relevant when considering the emotional content of stimuli, as its activation can be elicited even when individuals are engaged in a specific task if sufficiently emotionally intense distracters are presented (Iaria et al., 2008). This may depend on the fact that extremely appetitive or threatening stimuli override the current task, and demand the immediate reorienting of attention towards them, notwithstanding top-down attempts to persevere with the task at hand (Frank and Sabatinelli, 2012).

Regarding how specific EEG bands are implicated in mediating functional connectivity both between and within resting state networks, some studies report that, in the DMN, oscillations in the Alpha band (8–13 Hz; Atasoy et al., 2018; Marino et al., 2019a; Samogin et al., 2019) are the most prominent in promoting communication across all nodes, while high-frequency bands, such as the Gamma band (30–80 Hz; Atasoy et al., 2018), promote interaction between close node pairs (Samogin et al., 2019); in general, it is assumed that the higher the frequency, the closer the spatial range of the associated connectivity pattern (Jones et al., 2000; Kopell et al., 2000). In a recent study by Samogin et al. (2020), Gamma oscillations yielded the largest similarity with fMRI-derived measures of FC for some network seeds, such as the rTPJ in the VAN and the left temporo-parietal junction (lTPJ) in the LN. Furthermore, the Gamma band is particularly interesting because it is proposed to also reflect communication between task-relevant neuronal nodes (Jensen et al., 2007; Womelsdorf and Fries, 2007).

Keeping these findings in mind, as well as the fact that current literature on rs-FC and emotion dysregulation is still scarce, we aimed to investigate rs-FC in the six most widely analyzed RSNs in a sample of healthy women with high and low emotion dysregulation traits, by adopting an EEG paradigm to compute rs-FC. We decided to focus on a non-clinical sample of students because we aimed to avoid confounds that inevitably arise when studying a clinical or psychiatric population that is undergoing treatment, both concerning psychotherapy and psychopharmacology, as well as possibly confounding lifestyle variables. Moreover, the severity of symptoms of mental conditions that involve emotion dysregulation, such as BPD, typically peaks between the ages of 20 and 29 (Grant et al., 2008), and college-age students often report higher levels of psychological distress compared with non-student peers (Deasy et al., 2014). Within a student population, we decided to select a female-only sample for several reasons: the incidence of emotion dysregulation is higher in women, even starting at an early age (Bender et al., 2012); this holds also in young clinical samples (Wieckowski et al., 2020). Young girls are more prone than their male peers to engage in non-suicidal self-injury (NSSI) as an attempt to cope with negative emotions (Hawton and and-Harriss, 2008). Furthermore, Borderline Personality Disorder, which is the most extreme clinical manifestation of emotion dysregulation, is more prevalent in females (American Psychiatric Association, 2013). Another important reason for selecting females only is based on the substantial gender differences found in psychophysiological emotional responses (e.g., Bianchin and Angrilli, 2012). We aimed to reduce within-sample variance in emotion processing and regulation by working on the more homogenous sample represented by young women. Concerning the networks of interest, we concentrated on the VAN, given its role in the involuntary orienting of attention, particularly concerning emotion. Since, as earlier stated, the Alpha band is the most prominent resting state band, but the Gamma band was found to be important in the connectivity of our network of interest, we decided to focus on the Gamma band (30–50 Hz) and to use Alpha (8–13 Hz) as a control band.

## Materials and Methods

### Subject Selection

#### Rationale and Questionnaires Used

To conceptualize emotion dysregulation in a simple and objective manner, we used self-report questionnaires that are expected to capture facets of the construct that can be easily translated into a laboratory setting, while keeping at the same time enough efficacy to pinpoint the core features of this condition and account for its multidimensionality. Accordingly, we focused on the three main criteria applied in the DSM-5 for the diagnosis of BPD (American Psychiatric Association, 2013): difficulties in controlling anger, affective lability, and impulsive behavior. Therefore, we administered three questionnaires: (1) the ***Multidimensional Anger Inventory*** (MAI; Siegel, 1986) to assess anger and difficulties controlling it, which is a 38-item questionnaire scored on a 5-point Likert scale (from 1 = “Completely undescriptive of me” to 5 = “Completely descriptive of me”, with 7 items to be reverse scored). The MAI has proven to have a good internal consistency (Cronbach’s α = 0.88) and its subscales examine both subjective views of one’s own rage and the situations which elicit it. The subscales are the following: *Frequency*, *Duration*, *Magnitude*, *Anger-in*, *Anger-out*, *Guilt*, *Brood*, *Anger-discuss*, *Hostile outlook*, *Range of anger-eliciting situations*; (2) the ***Affective Lability Scales-18*** (ALS-18; Oliver and Simons, 2004), an 18-item questionnaire to assess instability in mood, which measures changeability among euthymia and four affective states: depression, elation, anxiety, and anger. Items are rated on a 4-point Likert scale (from 0 = “Very uncharacteristic of me”, to 3 = “Very characteristic of me”). The internal consistency of the instrument is good (Cronbach’s α = 0.95) and it comprises three subscales: *Anxiety/Depression*, *Depression/Elation*, and *Anger*; and (3) the ***UPPS-P Impulsive Behavior Scale*** (Lynam et al., 2006)to assess impulsivity. The UPPS-P has 59 items and five subscales which assess various manifestations of impulsive behavior: *Sensation Seeking*, *Lack of Premeditation*, *Lack of Perseverance*, *Positive Urgency*, and *Negative Urgency*. The items are rated on a 4-point Likert scale (from 1 = “Agree strongly” to 4 = “Disagree strongly”). We also administered the Balanced Inventory of Desirable Responding (BIDR-6; Paulhus, 1991) as a control scale to account for the impact of social desirability in the students’ responses.

#### Exclusion Criteria, Selection Strategy, and Sample Demographics

The questionnaires described in the above paragraphs were administered online to a sample of 422 female students of the University of Padova. We applied the exclusion criteria that are typically necessary to conduct an EEG experiment, such as a history of epilepsy, concussion, or other neurological problems, and ongoing treatment with psychotropic medication. We further excluded participants scoring over 2 standard deviations (SDs) above the normative mean score at the Impression Management subscale of the BIDR-6, which is the one most related to how people want to present themselves to others in a socially acceptable manner, correlating highly and positively with traditional lie scales and dissimulation measures (Paulhus, 2002). Two-hundred ninety-four students remained eligible for the study. To reduce data dimensionality and obtain a single score that takes account of the three questionnaires, we applied the Principal Component Analysis on the data in order to achieve two experimental groups: a Low Dysregulation (LD) group, comprising 25 subjects ranking below the 15th percentile, and a High Dysregulation (HD) group, comprising 25 subjects ranking above the 85th percentile. The two groups were balanced for age (*M*_HD_ = 22.64, *SD*_HD_ ± 2.12; *M*_LD_ = 22.60, *SD*_LD_ ± 1.63) and for lifestyle variables, such as daily caffeine and nicotine intake, hours of physical activity/week, and weekly consumption of alcohol. The demographic characteristics of the sample are summarized in Table 1.

**Table 1:** Socio-demographic characteristics of the sample.

	High dysregulation	Low dysregulation	Statistic
Age (in years, mean ± SD)	22.64 ± 2.12	22.60 ± 1.63	*t*_(48)_ = 0.07^†^
Physical activity
Yes	12 (48%)	9 (36%)	
No	13 (52%)	16 (64%)	*χ^2^* = 0.32^†^
Weekly hours of physical activity (mean ± SD)	4.08 ± 1.93	3.78 ± 1.2	*t*_(21)_ = 0.45^†^
Coffee intake
Yes	18 (72%)	13 (52%)	*χ^2^* = 1.36^†^
No	7 (28%)	12 (48%)	
Daily number of coffees (mean ± SD)	2.28 ±	1.85 ±	*t*_(29)_ = 1.46^†^
Alcohol use
Yes	18 (72%)	19 (76%)	*χ^2^* = 0 *(approx.)*^†^
No	7 (28%)	6 (24%)	
Weekly alcohol consumption (mean unit n. ± SD)	2.89 ± 2.49	2 ± 1	*t*_(35)_ = 1.41^†^
Drinking onset (meanagein years ± SD)	14.28 ± 1.13	15.84 ± 1.6	t_(35)_ = −3.44**
Cigarette smoking
Yes	6 (24%)	3 (12%)	*χ^2^* = 0.54^†^
No	19 (76%)	22 (88%)	
Daily cigarettes smoked (mean *n*. ± SD)	5.84 ± 6.05	7 ± 3.46	*t*_(7)_ = −0.37^†^
Smoking onset (mean age in years ± SD)	16.17 ± 3.6	18 ± 3	t_(7)_ = −0.80^†^
Illegal drug use (cannabis)
Yes	5 (20%)	1 (4%)	*χ^2^* = 1.7^†^
No	20 (80%)	24 (96%)	

Note: the six participants who answered “yes” reported only a sporadic use of cannabis (“rarely—almost never”). **p < 0.01; †: not significant (*p* > 0.05).

### Experimental Setting and EEG Recording

The study was conducted according to the guidelines of the Declaration of Helsinki and approved by the Ethics Committee of Psychology Area, University of Padova (protocol code 2989, date of approval 05/03/2019). As a first step, we asked the subjects to sign an informed consent form to participate in the research. Next, to ensure the correct attribution of the subjects to the experimental groups, given that the ALS-18 proved to be the most represented questionnaire in PC1 loadings, we asked the subjects in the laboratory to again fill in the ALS-18 prior to the EEG recording. The ALS-18 (Oliver and Simons, 2004) is an 18-item questionnaire, which is rated on a 4-point Likert scale (from 0 = “Very uncharacteristic of me” to 3 = “Very characteristic of me”) and comprises three subscales: Anxiety/Depression subscale, Depression/Elation, and an Anger subscale.

The subjects were then asked to sit in a comfortable chair while experimenters proceeded to fit them with an EEG elastic cap (ElectroCap) with 57 tin electrodes and seven additional external tin electrodes (on nasion, i.e., Nz, left and right external canthi, below left and right eyes, representing the EOG channels, plus the two mastoids) to record their spontaneous brain activity using a SynAmps amplifier (NeuroScan Labs, Sterling, USA) with 500 Hz sampling rate, bandwidth was set to 0–100 Hz (100 Hz low-pass antialiasing filter), 24 bit corresponding to 0.01 uV resolution. All channels were online referred to Cz.

After the montage was completed, the subject was asked to sit back and relax with her eyes open, the arms and legs uncrossed with the feet firmly placed on the floor and arms on the armrests of the chair. They were also asked to look in front of them, without visually exploring the room. No fixation cross was provided to avoid biasing their focus of attention on something specific. Since we were interested in assessing VAN connectivity, we decided to let participants’ minds wander freely without providing a fixation task. Resting state EEG activity was then recorded continuously for 5 min from each participant.

### EEG Signal Processing

We used an innovative EEG processing approach (Liu et al., 2017; Marino et al., 2019b) that allows performing seed-based connectivity analysis in the source space, as described by Samogin et al. (2020). This workflow is highly automated and consists of pre-processing the signal, with no subjective influence from the researcher (e.g., in choosing bad channels or in removing artifactual contributions), head model reconstruction, source localization of the cleaned EEG signal, and connectivity analysis.

First, in the pre-processing step, bad channels were detected, and biological artifacts were removed (Liu et al., 2017; Samogin et al., 2019). Bad channel detection was performed based on the values of two parameters: we calculated the minimum Pearson correlation between each channel signal and all signals from the other channels in the 1–50 Hz frequency range, and the noise variance in the 200–250 Hz frequency range, in which brain activity is considered negligible. Bad channels were considered those in which at least one of these two parameters was an outlier (average value + 4*SD; Liu et al., 2017) compared to the total distribution; the signal in these channels was reconstructed by interpolation of the neighboring channels using the FieldTrip toolbox (Oostenveld et al., 2011,^1^).

Next, EEGLab (Delorme and Makeig, 2004) was used to band-pass filter the EEG data in the 1–50 Hz band, and artifact removal was performed. Eye movement artifacts and noise from muscular activity were attenuated using Independent Component Analysis (ICA; Mantini et al., 2008). More in detail, a fast fixed-point ICA algorithm (FastICA^2^; Oja and Yuan, 2006) was used, and the noisy ICs were automatically identified using the procedure described by Liu and colleagues (Liu et al., 2017), in which the following parameters are considered: (a) correlation of the IC power with the power of the vEOG, hEOG and EMG signals; (b) the IC power spectrum fit against a 1/f function; and (c) the kurtosis of the IC time-course. The thresholds used for each parameter were set in accordance with previous studies (Mantini et al., 2009; De Pasquale et al., 2010; Liu et al., 2017), and only one parameter needed to be above its specific threshold for the IC to be considered artifactual (Samogin et al., 2020).

Since an individual structural MRI was not available for our participants, we used an MRI template, combined with the template electrode positions provided by the EEG system manufacturer, to build a standard head model. Source localization was performed using the exact low-resolution brain electromagnetic tomography algorithm (eLORETA; Pascual-Marqui et al., 2002) in a 6 mm homogeneous grid (Samogin et al., 2020).

Starting from the source localized EEG data, we focused on the signals from a set of seeds representative of commonly investigated RSNs, including the DMN, DAN, VAN, LN, SMN, and VN. Their coordinates were based on previous research (Samogin et al., 2019, 2020; Taberna et al., 2021). The MNI coordinates of the individual seeds that were used for FC analysis (Taberna et al., 2021) are listed in Table 2.

**Table 2:** MNI coordinates of the seeds we used for each network (Taberna et al., 2021).

Network	Seed	MNI coordinates		
Default Mode (DMN)	lAG	−32	−76	44
	rAG	57	−63	17
	PCC	−2	−50	30
	MPFC	−2	32	−10
DorsalAttention (DAN)	lIPS	−34	−48	44
	rIPS	33	−57	41
	lFEF	−32	−6	49
	rFEF	36	−19	44
VentralAttention (VAN)	rTPJ	60	−43	16
	rIFG	45	29	10
Language (LN)	lTPJ	−54	−33	−4
	lIFG	−47	14	1
Somatomotor (SMN)	lSMA	−1	−17	55
	lS1	−45	−17	49
	rS1	45	−17	49
	lS2	−42	−13	10
	rS2	42	−13	10
Visual (VN)	lhvV4	−27	−81	−13
	rhvV4	24	−82	−16
	ldV2	−44	−67	1
	rdV2	44	−73	−1

We computed the power spectrum of each ROI in the range (1–50 Hz), and then reconstructed the power spectrum of each RSN by averaging those constituting its ROIs. We extracted the power in the following bands: Delta (1–4 Hz), Theta (4–8 Hz), Alpha (8–13 Hz), Beta (13–30 Hz), and Gamma (30–50 Hz). Next, EEG connectivity was measured using Pearson correlations that were computed between the logarithmic-transformed signal-orthogonalized power time-courses and then transformed into z-values using the Fisher transform (De Pasquale et al., 2012; Hipp et al., 2012). We examined the EEG connectivity profiles between pairs of RSNs, for each frequency band. In particular, we computed inter-network connectivity as the average connectivity between all the possible pairs of ROIs belonging to two different networks (Newton et al., 2011). Frequency-specific inter-network connectivity values were averaged within the five frequency bands of interest (Delta, Theta, Alpha, Beta, and Gamma). For each frequency band and for each pair of RSNs, we then regressed out the difference in power from the connectivity values (Samogin et al., 2020).

### Statistical Analyses

On the connectivity matrices, Welch’s *t*-tests (Welch, 1947) were computed to assess differences regarding the average connectivity between different networks in the HD group compared with the LD group.

Moreover, Spearman’s rank correlation coefficients were calculated for each group between Alpha and Gamma power in the DMN and VAN with the second administration of the ALS-18 and its subscales as a measure of emotion dysregulation and mood instability. We computed Spearman’s rank correlation coefficients and not Pearson’s because, since the HD and LD represented two extreme groups, their questionnaire scores were not normally distributed (as measured with the Shapiro-Wilk normality test) and, when data violate the normality distribution assumption, the use of Spearman’s correlation is advised (Artusi et al., 2002). Multiple comparisons were corrected by means of False Discovery Rate (FDR) and the significance level was set to *p* < 0.05.

## Results

### Inter-network Connectivity Differences Between the Two Groups

Regarding the Alpha band (8–13 Hz), no significant between-group differences in inter-network connectivity emerged (see Table 3).

**Table 3:** Mean connectivity values, respective standard deviation (SD) by group, and *t*-test values comparing inter-network connectivity of the two groups on the Alpha band.

Network pair	High dysregulation group connectivity values *(mean ± SD)*	Low dysregulation group connectivity values *(mean ± SD)*	Welch’s *t* statistic	
DMN-DAN	0.062 ± 0.044	0.069 ± 0.069	−0.38^†^
DMN-VAN	0.076 ± 0.047	0.062 ± 0.057	−0.93^†^
DMN-LN	0.043 ± 0.041	0.066 ± 0.068	−1.45^†^
DMN-SMN	0.078 ± 0.039	0.084 ± 0.068	−0.41^†^
DMN-VN	0.10 ± 0.049	0.13 ± 0.076	−1.35^†^
DAN-VAN	0.099 ± 0.046	0.10 ± 0.061	−0.11^†^
DAN-LN	0.10 ± 0.042	0.12 ± 0.070	−1.39^†^
DAN-SMN	0.12 ± 0.049	0.10 ± 0.070	−1.32^†^
DAN-VN	0.054 ± 0.040	0.031 ± 0.068	−1.46^†^
VAN-LN	0.081 ± 0.042	0.088 ± 0.061	−0.46^†^
VAN-SMN	0.102 ± 0.043	0.104 ± 0.062	−0.13^†^
VAN-VN	0.050 ± 0.049	0.056 ± 0.058	−0.45^†^
LN-SMN	0.096 ± 0.039	0.10 ± 0.070	−0.44^†^
LN-VN	0.059 ± 0.042	0.046 ± 0.072	−0.80^†^
SMN-VN	0.060 ± 0.035	0.047 ± 0.062	−0.97^†^

*DMN, Default Mode Network; DAN, Dorsal Attention Network; VAN, Ventral Attention Network; LN, Language Network; SMN, Somatomotor Network; VN, Visual Network. †: not significant*.

**Figure 1:**
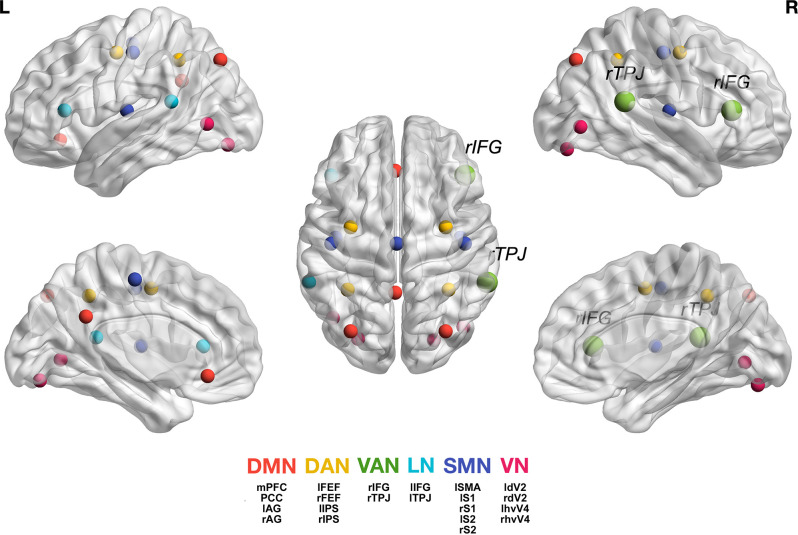
Graphic representation of the seeds we used for each network (Taberna et al., 2021). For the individual seed coordinates, see also Table 2. DMN (red), Default Mode Network; DAN (yellow), Dorsal Attention Network; VAN (green), Ventral Attention Network, with *rTPJ*, right temporo-parietal junction and *rIFG*, right inferior frontal gyrus; LN (light blue), Language Network; SMN (blue), Somatomotor Network; VN (magenta), Visual Network. The full names of the seeds are as follows: Posterior Cingulate Cortex (PCC), Medial Prefrontal Cortex (MPFC), Left Angular Gyrus (lAG); Right Angular Gyrus (rAG); Left Frontal Eye Field (lFEF), Right Frontal Eye Field (rFEF), Left Inferior Parietal Sulcus (lIPS), Right Inferior Parietal Sulcus (rIPS); Left Temporo-Parietal Junction (ITPJ), Left Inferior Frontal Gyrus (lIFG); Left Supplementary Motor Area (lSMA), Left Primary Somatosensory Cortex (lS1), Right Primary Somatosensory Cortex (rS1), Left Secondary Somatosensory Cortex (lS2), Right Secondary Somatosensory Cortex (rS2); Left Human Ventral Visual 4 Area (lhvV4), Right Human Ventral Visual 4 Area (rhvV4), Left Dorsal Visual 2 Area (ldV2), Right Dorsal Visual 2 Area (rdV2). Figure generation was obtained with BrainNet Viewer version 1.7 (Xia et al., 2013).

In the Gamma band (30–50 Hz), however, the HD group showed stronger connectivity between the VAN and all other networks compared with the LD group. Figure 1 shows the location of all analyzed seeds and reports the VAN was bigger as it was more activated and connected with most of the other seeds.

The LD group had stronger connectivity between the DAN and LN (*t*_(48)_ = −2.8, *p* < 0.01) and between the LN and SMN (*t*_(48)_ = −2.59, *p* < 0.05). All results for the Gamma band are summarized in Table 4.

**Table 4:** Mean connectivity values, respective standard deviation (SD) by group and *t* test values comparing inter-network connectivity of the two groups on the Gamma band.

Network pair	High Dysregulation group connectivity values *(mean ± SD)*	Low Dysregulation group connectivity values *(mean ± SD)*	Welch’s *t* statistic	
DMN-DAN	0.068 ± 0.048	0.044 ± 0.067	1.42^†^
**DMN-VAN**	**0.08 ± 0.062**	**0.001 ± 0.063**	4.47***
DMN-LN	0.058 ± 0.057	0.083 ± 0.066	−1.44^†^
DMN-SMN	0.069 ± 0.51	0.060 ± 0.68	0.53^†^
DMN-VN	0.046 ± 0.046	0.045 ± 0.052	0.10^†^
**DAN-VAN**	**0.098 ± 0.069**	
**−0.01 ± 0.076**	5.7***
DAN-LN	0.057 ± 0.073	0.12 ± 0.086	−2.8**
DAN-SMN	0.043 ± 0.061	0.034 ± 0.081	0.44^†^
DAN-VN	0.028 ± 0.046	0.035 ± 0.061	−0.43^†^
**VAN-LN**	**0.087 ± 0.082**	**0.027 ± 0.068**	2.8**
**VAN-SMN**	**0.12 ± 0.073**	**−0.006 ± 0.08**	6.04***
**VAN-VN**	**0.11 ± 0.08**	**0.027 ± 0.067**	4.28***
LN-SMN	0.08 ± 0.073	0.13 ± 0.08	−2.59*
LN-VN	0.073 ± 0.60	0.066 ± 0.63	0.40^†^‥
SMN-VN	0.054 ± 0.052	0.051 ± 0.063	0.22^†^‥

DMN, Default Mode Network; DAN, Dorsal Attention Network; VAN, Ventral Attention Network; LN, Language Network; SMN, Somatomotor Network; VN, Visual Network. *p < 0.05; **p < 0.01; ***p < 0.001; ^†^, not significant.

### Correlations Between Connectivity Measures, Network Power, and Affective Lability (ALS-18)

**Figure 2:**
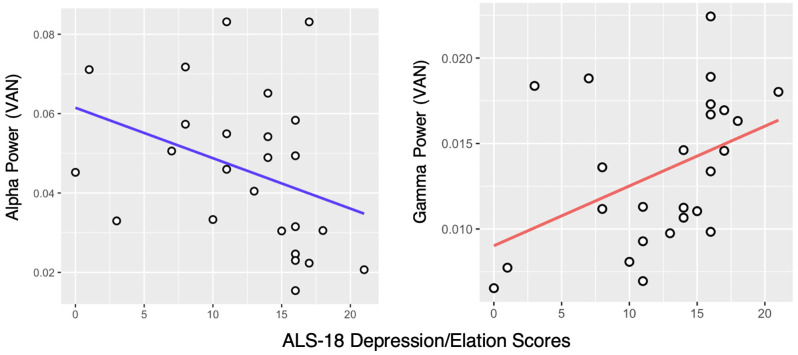
Correlation between the ALS-18 Depression/Elation scale scores and Alpha and Gamma power in the Ventral Attention Network (VAN) in the High Dysregulation group. Spearman’s rank correlation coefficients are *ρ* = −0.45, *p*_FDR_ < 0.05 and *ρ* = 0.47, *p*_FDR_ < 0.05, respectively.

Alpha band (8–13 Hz) power in the VAN was negatively correlated with affective instability as measured with the ALS-18, both regarding total score (*ρ* = −0.52, *p*_FDR_ < 0.01) and the Depression/Elation subscale (*ρ* = −0.45, *p*_FDR_ < 0.05; Figure 2) in the HD group, but not in the LD group. Alpha power in the DMN was also negatively correlated with the ALS-18 total score (*ρ* = −0.4, *p*_FDR_ < 0.05) in the HD group. No significant correlations were found in the LD group.

Looking at the Gamma band (30–50 Hz), a positive correlation was found between Gamma power in the VAN and the Depression/Elation subscale of the ALS-18, again in the HD group only (*ρ* = 0.47, *p*_FDR_ < 0.05; Figure 2). In the LD group, no significant correlations were found.

## Discussion

The study of brain activity during the resting state, and particularly its functional organization in networks, is one of the most thoroughly investigated topics in neuroscience in recent years. However, when looking at the construct of emotion dysregulation, studies are still scarce and have so far yielded inconsistent results. Most studies have focused on active emotion regulation during specific tasks, a setting that is not suitable for studying resting state activity; additionally, since tasks and paradigms vary greatly among studies, some concerns have been raised regarding the appropriateness of emotion regulation as a construct to be the focus of scientific, systematic investigation (Beauchaine, 2015). Pathological emotion dysregulation is a key domain which is altered in almost all severe psychiatric diseases, including all anxiety and mood disorders, some psychotic subgroups, but also several personality disorders such as those in cluster B (American Psychiatric Association, 2013). Exploring the basic structured features of emotion dysregulation in a healthy sample would provide hints for both prevention and early interventions before a severe disorder appears. While most of the psychiatric literature focused on the DMN, we hypothesized that the VAN, a network ignited by emotion-related attention to biologically relevant stimuli, including the internal ones, would have shown the greatest activation and dominance in individuals with emotion regulation problems. Interestingly, the VAN includes key regions in the right hemisphere (rIFC, rTPJ) tightly connected with a larger network comprising also right insula, rACC and right limbic structures (amygdala, thalamus, basal ganglia) known to be involved in controlling and regulating emotional processes and, although limited, past research showed the key emotion-regulating role of the VAN (Iaria et al., 2008; Frank and Sabatinelli, 2012) also in depressed individuals (Mao et al., 2020).

A 2016 meta-analysis by Visintin and colleagues conducted on seven neuroimaging studies reported alterations in DMN regions in BPD patients compared with healthy controls. In particular, BPD patients are characterized by increased activity in the precuneus both at rest and during emotional processing, and reduced activity and smaller gray matter volume in the lateral temporal complex (Visintin et al., 2016). More recently, a study by Lei and colleagues found increased rs-FC of the left anterior cingulate cortex (ACC) with the right middle frontal gyrus, decreased rs-FC of the left ACC with the left middle temporal gyrus, and of both the left and the right ACC with the corpus callosum in BPD patients compared with healthy controls. FC metrics for the left ACC also correlated negatively with cognitive emotion regulation and depressive symptoms in BPD patients (Lei et al., 2019). Another study using a seed-based approach computed after the measurement of the Intrinsic Connectivity Contrast (ICC) on all the voxels in the brain allowed us to identify two seeds in which connectivity was stronger in BPD compared with healthy controls: the caudate nucleus, which in patients showed greater FC with the ACC, the left and right ventral striatum, the medial prefrontal cortex, the paracingulate gyrus, and the supplementary motor area; and the left insula, which was hyperconnected with the midcingulate and the dorsal ACC, the left and right orbitofrontal cortex, both left and right inferior parietal lobule and the right precentral gyrus (Sarkheil et al., 2020).

The present research aimed to disentangle the role of rs-FC of widely studied RSNs in a community sample of healthy women with high (HD group) and low (LD group) traits of emotion dysregulation. More in detail, we analyzed connectivity patterns in the DMN, the DAN, the VAN, the LN, the SMN, and the VN. We, therefore, adopted an EEG paradigm, and we expected the Gamma activity (30–50 Hz) to better reflect cortical activity related to processes activated during the resting state, especially in the VAN (ElShafei et al., 2020). At the same time, Alpha (8–13 Hz) served as a control band given the resting state condition that was the focus of the study, and Alpha was found to be the most prominent rhythm at rest (Samogin et al., 2019). We considered the VAN, the network most involved in the attentional biases towards emotional stimuli, especially on personally relevant biographical ones, which have been found in individuals that suffer from emotional disturbances, such as patients with a diagnosis of BPD (Kaiser et al., 2016).

Indeed, the VAN was the most connected with the other five analyzed RSNs in the HD group compared to the LD group, in the Gamma band, but not in the Alpha band. In addition to mediating connectivity between the VAN and all the other RSNs, Gamma power in the VAN of the HD group was also positively correlated with measures of affective lability (the ALS-18 questionnaire), especially with the subscale measuring shifts between states of depression and elation. In the HD group, we found an additional negative correlation between the Alpha power of the VAN and affective instability, which was not replicated in the LD group. These findings point towards a pattern of excessive activation/synchronization of the VAN in individuals with high traits of emotion dysregulation.

The VAN plays a relevant role in the automatic reorienting of attention towards an emotional stimulus, which eventually leads to overriding top-down control that would demand the subject to be engaged in other activities (Iaria et al., 2008; Frank and Sabatinelli, 2012). Our finding suggests that an exaggerated focus on internal emotional content, particularly if negative or rapidly shifting between negative and positive prompts, might be reflected in an increased connectivity between the VAN and the other RSNs. This is especially relevant in people with difficulties in appropriately regulating emotional responses. Indeed, Gamma activity in the VAN was increased during bottom-up attentional control following distracting stimuli (ElShafei et al., 2020), even in the primate brain following bottom-up attentional capture (Buschman and Miller, 2007). Furthermore, the Gamma band has been shown to mediate communication between task-relevant nodes (Jensen et al., 2007; Womelsdorf and Fries, 2007). The fact that we found greater Gamma connectivity between the VAN and the other RSNs in a resting state condition is peculiar to this regard as this result points to an involuntary capture of these subjects by their emotional, internal turmoil—a process that involves the entire brain. This finding was further corroborated by a significant negative association between Alpha power in the DMN and shifts in mood, suggesting a possibly protective role of Alpha band oscillations in the DMN as a general inhibitory mechanism that dampens emotional dysregulation and mood swings. Indeed, Alpha/Theta neurofeedback (A/T NF), which aims to increase Alpha and Theta band activity to induce a state of relaxation (Egner et al., 2002), has been found to increase connectivity in the DMN, which was in turn associated with an increase in mentalization skills—the set of abilities needed to understand and give meaning to the inner mental and affective states of both oneself and others (Fonagy and Bateman, 2008; Imperatori et al., 2017). Further support to this interpretation is provided by the observation that self-focused mentalization skills are associated with efficient emotion regulation, and they allowed to predict both adaptive and maladaptive emotion regulation independently of age, gender, and native language (Schwarzer et al., 2021). Moreover, since the late 1980s A/T NF has been successfully employed to increase Alpha rhythm amplitudes in resting state EEG and reduce self-reported depressive symptoms in alcoholic patients (Peniston and Kulkosky, 1989), again pointing to a protective effect of this band synchronization in populations with emotional difficulties.

While no overall significant differences in Gamma and Alpha activity were found between the groups, the associations between Gamma power and affective lability that characterized the HD group only, and the negative associations that we found between affective lability and Alpha power in the VAN and the DMN, again in the HD group only, point towards an association between a pattern of greater activation in this network in general and emotion dysregulation even at a trait level. This association may further lead to finding larger, significant differences in Alpha and Gamma power if, for example, clinical populations vs. healthy controls will be studied in future research.

Our study shed some light on the possible neural mechanisms underlying emotion dysregulation in a resting state condition and highlighted how research should not only focus on the DMN but also consider the global architecture of the brain, by measuring all the RSNs and how they interact together. Involuntary attentional processes, additionally, are revealed to be important even when subjects are not actively and explicitly engaged in a task, suggesting that certain populations could have difficulties in actually “resting” their mind during resting state, and are instead excessively focused on internally generated emotional prompts that distract them and catch their attention. As a future direction, this could also be probed, for instance, in depressed patients with particularly impairing rumination symptoms, or in other mood or anxiety disorders, or in Obsessive Compulsive Disorder (OCD; American Psychiatric Association, 2013).

Among the limitations of our research, concerning the EEG analysis, the computed head model was based on electrode positioning templates and standard anatomy, which partially limited the accuracy of source localization. Future studies aiming to replicate or broaden these findings could be based on higher-density EEG recordings, for which the exact positioning of the EEG sensors (Taberna et al., 2019) as well as an individual head model for each subject should be available (Taberna et al., 2021). Moreover, we only concentrated on a female population; it would be interesting to assess gender differences in rs-FC by also including males that exhibit trait emotion dysregulation, a population which is largely neglected in the existing literature. Another limitation lies with the fact that our sample was very specific, and not clinical. While we insist on affirming the methodological advantage that studying healthy samples has, it would still be worthwhile to compare rs-FC measures in clinical populations, such as BPD patients, with those of participants exhibiting high traits of emotion dysregulation, to test whether and how they differ in connectivity patterns. Another possible approach based on a longitudinal design should allow distinguishing participants with high trait emotion dysregulation that develop a clinical disorder in later years, from those who do not convert, also considering the possible role of altered brain network dynamics as biological markers of disease vulnerability. Notably, our research highlights the importance of studying emotion dysregulation even regarding its trait form, in non-clinical populations. Expanding our findings with more precise source localization may offer additional information on the identification of biomarkers of emotion dysregulation in young, healthy individuals, and contribute to the development of tailored psycho-educational interventions to improve healthy management of emotion and general wellbeing in the adult population.

## Data Availability Statement

Data collected in this study can be requested to the corresponding author upon justified request for academic purposes only.

## Ethics Statement

The studies involving human participants were reviewed and approved by COMITATO ETICO DELLA RICERCA PSICOLOGICA AREA 17 - Dipartimenti/Sezione di Psicologia Università degli Studi di Padova via Venezia 8, 35131 Padova (Italy). FAX. +39 49 827 6600. E-mail: comitato.etico.area17@unipd.it. The patients/participants provided their written informed consent to participate in this study.

## Author Contributions

FF, CS, and AA: conceptualization and project development. FF data collection, data analysis and draft preparation. MM: data analysis. All authors contributed to the article and approved the submitted version.

## Funding

The present work was also carried out within the scope of the research program Dipartimenti di Eccellenza (art.1, commi 314-337 legge 232/2016), which was supported by a grant from MIUR to the Department of General Psychology, University of Padua. The PhD fellowship of FF was supported by the same grant. MM was funded by the Research Foundation Flanders (FWO; postdoctoral fellowship 1211820N). CS was funded by the Italian Ministry of Education and Research (PRIN 2017 grant, project n. 20178 NNRCR_003).

## Footnotes

^1^ http://www.fieldtriptoolbox.org/

^2^ http://research.ics.aalto.fi/ica/fastica
